# Mortality prediction of patients in intensive care units using machine learning algorithms based on electronic health records

**DOI:** 10.1038/s41598-022-11226-4

**Published:** 2022-05-03

**Authors:** Min Hyuk Choi, Dokyun Kim, Eui Jun Choi, Yeo Jin Jung, Yong Jun Choi, Jae Hwa Cho, Seok Hoon Jeong

**Affiliations:** 1 Department of Laboratory Medicine and Research Institute of Bacterial Resistance, Gangnam Severance Hospital, Yonsei University College of Medicine, 211 Eonju-ro, Gangnam-gu, Seoul, 06273 South Korea; 2 Department of Statistics and Data Science, Yonsei University, Seoul, South Korea; 3 Department of Internal Medicine, Gangnam Severance Hospital, Yonsei University College of Medicine, Seoul, South Korea

**Keywords:** Prognosis, Machine learning

## Abstract

Improving predictive models for intensive care unit (ICU) inpatients requires a new strategy that periodically includes the latest clinical data and can be updated to reflect local characteristics. We extracted data from all adult patients admitted to the ICUs of two university hospitals with different characteristics from 2006 to 2020, and a total of 85,146 patients were included in this study. Machine learning algorithms were trained to predict in-hospital mortality. The predictive performance of conventional scoring models and machine learning algorithms was assessed by the area under the receiver operating characteristic curve (AUROC). The conventional scoring models had various predictive powers, with the SAPS III (AUROC 0.773 [0.766–0.779] for hospital S) and APACHE III (AUROC 0.803 [0.795–0.810] for hospital G) showing the highest AUROC among them. The best performing machine learning models achieved an AUROC of 0.977 (0.973–0.980) in hospital S and 0.955 (0.950–0.961) in hospital G. The use of ML models in conjunction with conventional scoring systems can provide more useful information for predicting the prognosis of critically ill patients. In this study, we suggest that the predictive model can be made more robust by training with the individual data of each hospital.

## Introduction

Intensive care unit (ICU) is a special setting in which patients have a higher baseline severity at the time of admission and hospital-associated infections occur more frequently than in general wards^1,2^. Due to the high mortality rate of ICU patients, several scoring systems have been used to predict patient’s prognosis^3,4^. Conventional scoring systems such as the Assessment and Chronic Health Evaluation (APACHE) II and III, the Simplified Acute Physiology Score (SAPS) II and III, the Mortality Probability Model (MPM_0_) II and III, and the Sequential Oran Failure Assessment (SOFA) are moderately accurate in predicting individual mortality^1,3–8^. The Pitt bacteremia score has also been shown to predict the prognosis even in patients with nonbacterial infections^9^.

These scoring systems have the advantage of standardizing studies and allowing the comparison of quality of care between ICUs. However, the prognostic predictive power of conventional models may vary depending on the characteristics of the hospitals in which they are to be applied, such as the demographic composition of inpatients, underlying comorbidities, and the percentage of surgical or medical admissions^10–12^. In addition, since conventional scoring systems were established and validated at specific points in time, predictive performance can deteriorate over time due to temporal and social changes, including an extension of life expectancy, improvements in the public health environment, and the emergence of new diseases^10–13^.

To improve the predictive model for ICU inpatients, it is necessary to prepare a new strategy that can be easily updated periodically to include the latest clinical data and to reflect the local features of each hospital^14^. The growth of automated electronic health records (EHRs) has provided an opportunity to extract a large amount of clinical information for improving the performance of established models. Additionally, machine learning (ML) techniques are being implemented in various ways for diagnosis and prognosis prediction in the field of clinical medicine including critical care medicine^14–18^. Comprehensive data analysis through ML can leverage the vast number of variables already inputted into EHRs to prevent overfitting and facilitate the development of continuously updatable prediction models^14^.

The purpose of this study was to show the importance of an updatable prognostic prediction model that can reflect the characteristics of individual centers. We compared the predictive models of ICU inpatients with conventional scoring systems and ML algorithms. Furthermore, we also evaluated the conventional models by year and hospital and, through ML algorithms, investigated variables that mutually affected the prognosis of patients admitted to the ICUs.

## Methods

### Study population and data collection

To determine a ML and statistical analysis approach based on local EHR data for mortality prediction, we extracted data from all adult patients (age ≥ 18 years) admitted to medical, surgical, and cardiovascular ICUs from 2006 to 2020 using the Severance Clinical Research Analysis Portal [EHR data collection program with information since 2006 of two university hospitals in South Korea. As of October 2021, this portal contains the data of over four million patients in Severance Hospital (hospital S) and over two million patients in Gangnam Severance Hospital (hospital G) and is continuously updated in real-time with only a short delay]. To compare the differences in the modeling fit according to hospital characteristics, data were collected from two different university hospitals [hospital S and hospital G, tertiary university hospitals with more than 2000 (including more than 130 adult ICU beds) and 800 beds (including more than 40 adult ICU beds), respectively] during the study period.

Patient-level data were collected, including demographics, underlying comorbidities, date of first ICU admission, and date of death. To obtain the worst values within the first 24 h of each ICU stay, both maximum and minimum values of vital signs and laboratory test results were extracted. The use of mechanical ventilation, vasopressors, and antibiotics within the first 24 h of admission to the ICUs was investigated, and the lowest Glasgow Coma Scale and total urine output per day were also extracted. The site of infection were defined according to the criteria proposed by the Centers for Disease Control and Prevention and a previous study^19,20^. Predictive models were developed by selecting in-hospital mortality as the outcome of interest.

All processes of this study were in accordance with the ethical standards of the institutional and national research committee and with the 1964 Helsinki declaration and comparable ethical standards. The study was approved by the Institutional Review Board (approval no.: 2021–0575–001) of Yonsei University Health System (Seoul, Republic of Korea), which waived the requirement for written informed consent because of the retrospective nature of this study.

### Model development

Before the modeling, the type of admission, sex, type of admitted ICU, first site of bacterial infection, antibiotic use, and underlying comorbidities were treated as categorical values. In addition, continuous variables were standardized, and missing values were imputed using the median. A total of 70 features were included in the machine learning models.

**Figure 1:**
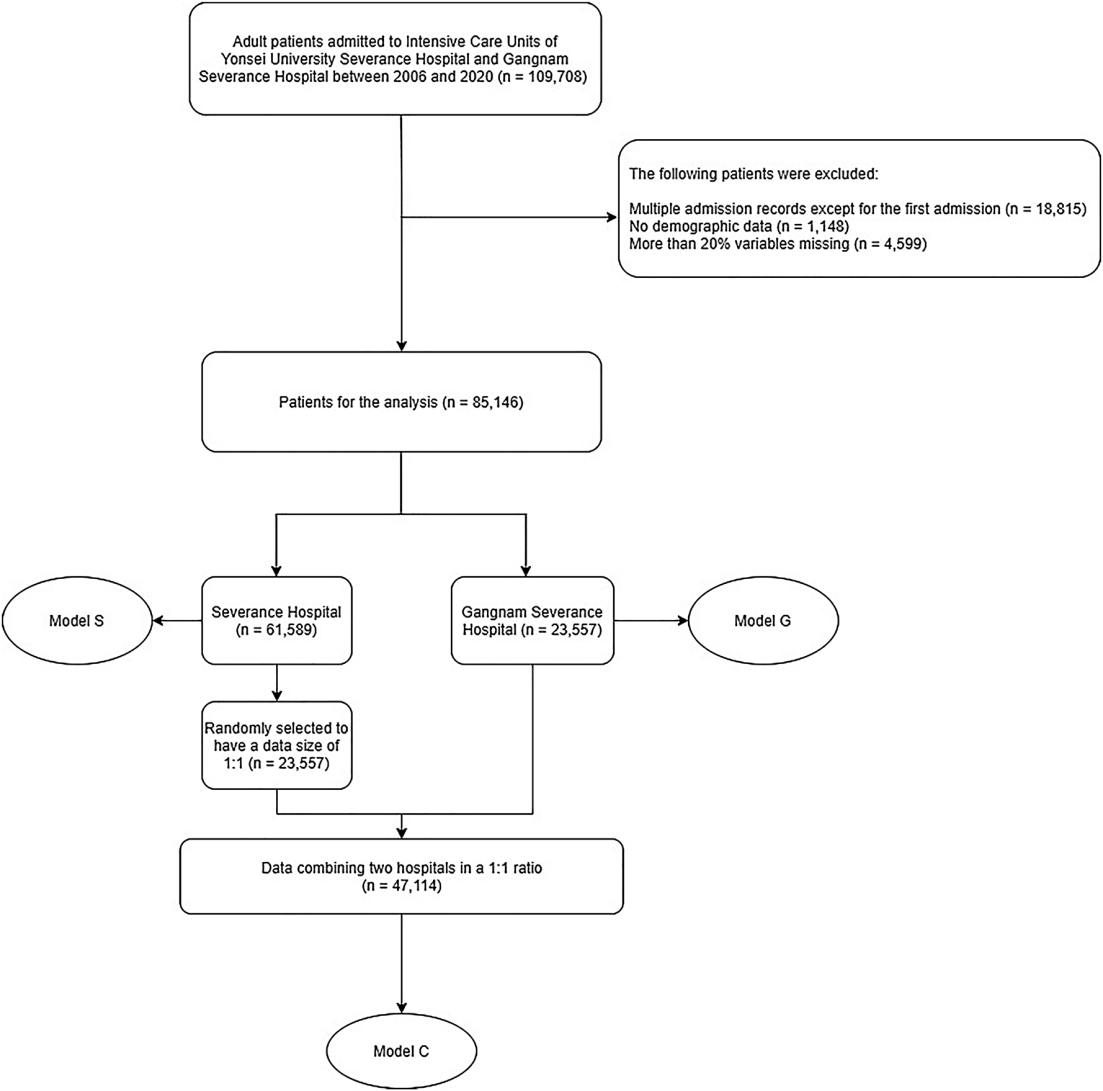
Flowchart depicting steps in obtaining the dataset.

The cohort C dataset included a random sampling of cases from hospital S with the same size as cases in hospital G, followed by a pooling of cases from both hospitals. Cohort S and cohort G included datasets from hospital S and hospital G patients, respectively (Fig. 1). The dataset was randomly split for all models. Eighty percent of the cases were used to train the scoring systems, and 20% were left as an internal test set for model evaluation. In addition, the model selected by training with cohort S was externally validated with cohort G and vice versa.

Candidate models were trained using K-nearest neighbor (KNN), decision tree (DT), random forest (RF), extreme gradient boosting (XGBoost), light gradient boosting (LightGBM), support vector machine (SVM) and artificial neural network (ANN) algorithms. ANN models were constructed with three layers with one input layer, two hidden layers (with 128 nodes applied), and one output layer. Models were trained to use the Root Mean Square Propagation algorithm to optimize the cross-entropy loss. For each model, hyperparameter tuning was randomly performed through grid search and fivefold cross-validation to find the optimal model while preventing overfitting. Each model with the highest area under the receiver operator characteristic curve (AUROC) and the 95% confidence intervals (CIs) were generated using bootstrapping^14,21^. We also used the F1 score (the harmonic mean of precision and recall), accuracy, and area under the precision-recall curve (AUPRC) as our secondary performance metrics for model comparison, as they are more informative for predicting in-hospital mortality^15,22,23^. To explain the model, we visualized feature importance rankings as feature weights, mean gain, and coverage for the XGBoost models and Shapley additive explanation (SHAP) summary dot plots for the LightGBM models^24^. We also presented calibration plots to illustrate each model’s calibration of consistency between observed and estimated in-hospital mortality.

ML models were analyzed using Python programming software version 3.7.12 (Python software foundation, http://www.python.org/) with the scikit-learn version 0.22.2 and TensorFlow version 2.6.0 libraries.

### Statistical analysis

All variables were assessed by the Shapiro–Wilk test to evaluate the normality of the distributions, and admission variables are described using numbers and percentages for categorical variables, medians, and interquartile ranges (IQRs) for continuous variables. Differences were tested with Fisher's exact test for qualitative data and with the Mann–Whitney U test for quantitative data. All reported *p* values were 2-sided, and statistical significance was assumed if *P* < 0.05. Statistical analyses were performed using R statistical software version 4.1 (R Studio, Inc., http://www.r-project.org/).

### Ethical approval

The study was approved by the Institutional Review Board (approval no.: 2021–0575–001) of Yonsei University Health System (Seoul, Republic of Korea), which waived the requirement for written informed consent because of the retrospective nature of this study.

## Results

Figure 1 is a flowchart showing the dataset selection process for modeling. During the study period, 109,708 patients were admitted to the medical, surgical, and cardiovascular ICUs of hospital S and hospital G. After excluding multiple admissions except for the first admission, patients without demographic data, and patients missing more than 20% of the variables, a total of 85,146 patients were included in the study.

The clinical and demographic characteristics are described in Table 1. The median age of the patients was 67 years (IQR, 57–74 years) in hospital S and 65 years (IQR, 53–75 years) in hospital G. There were statistically significant differences in all variables between patients with and without in-hospital mortality at hospital S. Similarly, in hospital G, all variables were significantly different between the two groups except for sex, history of cerebrovascular disease, and history of diabetes mellitus. In both cohorts, patients with in-hospital death were older, were more likely to be admitted for medical than surgical reasons, had higher scores on the conventional prediction model and the Charlson comorbidity index than patients without in-hospital death. The in-hospital mortality rates of hospital S and hospital G were 11.8% and 17.4%, respectively, which were significantly different (*P* < 0.001). Additionally, the scores of all conventional prediction models except MPM0 were significantly higher in hospital G than in hospital S (*P* < 0.001).

**Table 1:** Baseline characteristics and physiological variables obtained within 24 h of ICU admission.

Admission variables (obtained within 24 h of ICU admission)	Hospital S				Hospital G				*P*†
	Overall (N = 61,589)	No hospital mortality (N = 54,313)	Hospital mortality (N = 7276)	P*	Overall (N = 23,557)	No hospital mortality (N = 19,458)	Hospital mortality (N = 4099)	P*	
Age, years	67 [57–74]	66 [57–74]	67 [56–76]	< 0.001	65 [53–75]	64 [52–74]	70 [58–78]	< 0.001	< 0.001
Sex				0.004				0.174	< 0.001
Female	22,744 (36.9%)	19,944 (36.7%)	2800 (38.5%)		9034 (38.3%)	7501 (38.5%)	1533 (37.4%)		
Male	38,845 (63.1%)	34,369 (63.3%)	4476 (61.5%)		14,523 (61.7%)	11,957 (61.5%)	2566 (62.6%)		
Types of admission				< 0.001				< 0.001	< 0.001
Medical	39,560 (64.2%)	34,232 (63.0%)	5328 (73.2%)		11,365 (48.2%)	9073 (46.6%)	2292 (55.9%)		
Surgical	22,029 (35.8%)	20,081 (37.0%)	1948 (26.8%)		12,192 (51.8%)	10,385 (53.4%)	1807 (44.1%)		
Year of admission				< 0.001				< 0.001	0.033
2006–2010	16,531 (26.8%)	14,422 (26.6%)	2109 (29.0%)		6379 (27.1%)	5322 (27.4%)	1057 (25.8%)		
2011–2015	20,945 (34.0%)	18,371 (33.8%)	2574 (35.4%)		8179 (34.7%)	6637 (34.1%)	1542 (37.6%)		
2016–2020	24,113 (39.2%)	21,520 (39.6%)	2593 (35.6%)		8999 (38.2%)	7499 (38.5%)	1500 (36.6%)		
**Conventional scoring systems**									
APACHE II	13 [10–17]	13 [10–16]	19 [13–26]	< 0.001	16 [11–21]	15 [10–19]	22 [18–28]	< 0.001	< 0.001
APACHE III	55 [47–65]	54 [46–63]	74 [57–96]	< 0.001	60 [50–74]	57 [48–69]	79 [66–95]	< 0.001	< 0.001
SAPS II	34 [28–42]	33 [27–40]	48 [36–64]	< 0.001	37 [29–48]	35 [27–44]	52 [42–64]	< 0.001	< 0.001
SAPS III	47 [40–55]	46 [40–53]	59 [50–70]	< 0.001	51 [43–61]	49 [42–57]	65 [56–74]	< 0.001	< 0.001
MPM0 II	2 [2–2]	2 [2–2]	2 [1–3]	< 0.001	2 [1, 2]	1 [1, 2]	2 [1–3]	< 0.001	< 0.001
MPM0 III	2 [2–3]	2 [2, 3]	2 [1–3]	< 0.001	2 [1, 2]	2 [1, 2]	2 [2–4]	< 0.001	< 0.001
SOFA	4 [2–8]	4 [2–7]	7 [4–11]	< 0.001	6 [3–9]	6 [2–9]	10 [7–12]	< 0.001	< 0.001
Pitt bacteremia score	1 [0–4]	1 [0–4]	2 [0–5]	< 0.001	3 [1–5]	3 [1–4]	5 [3–7]	< 0.001	< 0.001
Underlying comorbidities									
Charlson comorbidity index	5 [4–6]	5 [4–6]	5 [4–7]	< 0.001	5 [3–6]	4 [3–6]	5 [4–7]	< 0.001	< 0.001
Cancer	5137 (8.3%)	3101 (5.7%)	2036 (28.0%)	< 0.001	4239 (18.0%)	3002 (15.4%)	1237 (30.2%)	< 0.001	< 0.001
Cerebrovascular diseases	14,373 (23.3%)	13,433 (24.7%)	940 (12.9%)	< 0.001	4533 (19.2%)	3716 (19.1%)	817 (19.9%)	0.226	< 0.001
Diabetes mellitus	16,696 (27.1%)	15,126 (27.8%)	1570 (21.6%)	< 0.001	4086 (17.3%)	3391 (17.4%)	695 (17.0%)	0.482	< 0.001
Hypertension	28,407 (46.1%)	26,538 (48.9%)	1869 (25.7%)	< 0.001	5406 (22.9%)	4677 (24.0%)	729 (17.8%)	< 0.001	< 0.001
Chronic pulmonary diseases	1832 (3.0%)	1364 (2.5%)	468 (6.4%)	< 0.001	541 (2.3%)	332 (1.7%)	209 (5.1%)	< 0.001	< 0.001
Hemiplegia	2041 (3.3%)	1900 (3.5%)	141 (1.9%)	< 0.001	1650 (7.0%)	1500 (7.7%)	150 (3.7%)	< 0.001	< 0.001
Liver diseases	2080 (3.4%)	1235 (2.3%)	845 (11.6%)	< 0.001	1248 (5.3%)	922 (4.7%)	326 (8.0%)	< 0.001	< 0.001
Myocardial infarction	11,682 (19.0%)	10,888 (20.0%)	794 (10.9%)	< 0.001	2886 (12.3%)	2482 (12.8%)	404 (9.9%)	< 0.001	< 0.001
Renal diseases	3462 (5.6%)	2798 (5.2%)	664 (9.1%)	< 0.001	1367 (5.8%)	981 (5.0%)	386 (9.4%)	< 0.001	0.313
Ulcer	1168 (1.9%)	926 (1.7%)	242 (3.3%)	< 0.001	485 (2.1%)	370 (1.9%)	115 (2.8%)	< 0.001	0.131
Transplantation	766 (1.2%)	265 (0.5%)	501 (6.9%)	< 0.001	203 (0.9%)	153 (0.8%)	50 (1.2%)	0.008	< 0.001
Ventilator use	10,537 (17.1%)	8895 (16.4%)	1642 (22.6%)	< 0.001	5957 (25.3%)	4745 (24.4%)	1212 (29.6%)	< 0.001	< 0.001
Vasopressor use	21,448 (34.8%)	18,089 (33.3%)	3359 (46.2%)	< 0.001	11,708 (49.7%)	8439 (43.4%)	3269 (79.8%)	< 0.001	< 0.001
Cardiac arrest	809 (1.3%)	694 (1.3%)	115 (1.6%)	0.038	1228 (5.2%)	414 (2.1%)	814 (19.9%)	< 0.001	< 0.001
**Bacterial infection on ICU admission**									
Site of infection				< 0.001				< 0.001	< 0.001
Multiple sites	843 (1.4%)	108 (0.2%)	735 (10.1%)		275 (1.2%)	906 (4.7%)	529 (12.9%)		
Lungs	428 (0.7%)	283 (0.5%)	145 (2.0%)		1435 (6.1%)	383 (2.0%)	238 (5.8%)		
Bloodstream	251 (0.4%)	119 (0.2%)	132 (1.8%)		183 (0.8%)	110 (0.6%)	73 (1.8%)		
Urinary tract	503 (0.8%)	363 (0.7%)	140 (1.9%)		621 (2.6%)	383 (2.0%)	238 (5.8%)		
CNS	3 (0.0%)	0 (0.0%)	3 (0.0%)		0 (0.0%)	165 (0.8%)	110 (2.7%)		
Abdomen	27 (0.0%)	19 (0.0%)	8 (0.1%)		156 (0.7%)	103 (0.5%)	53 (1.3%)		
None	59,521 (96.6%)	53,418 (98.4%)	6103 (83.9%)		20,887 (88.7%)	17,791 (91.4%)	3096 (75.5%)		
Antibiotic use at ICU admission (may be multiple)	28,255 (45.9%)	22,754 (41.9%)	5501 (75.6%)	< 0.001	16,867 (71.6%)	13,110 (67.4%)	3757 (91.7%)	< 0.001	< 0.001
3rd-generation cephalosporins	6788 (11.0%)	4947 (9.1%)	1841 (25.3%)	< 0.001	5649 (24.0%)	4490 (23.1%)	1159 (28.3%)	< 0.001	< 0.001
4th-generation cephalosporins	450 (0.7%)	30 (0.1%)	420 (5.8%)	< 0.001	591 (2.5%)	296 (1.5%)	295 (7.2%)	< 0.001	< 0.001
Beta lactam/beta lactamase inhibitors	8744 (14.2%)	5646 (10.4%)	3098 (42.6%)	< 0.001	6362 (27.0%)	4401 (22.6%)	1961 (47.8%)	< 0.001	< 0.001
Carbapenems	2300 (3.7%)	587 (1.1%)	1713 (23.5%)	< 0.001	2524 (10.7%)	1409 (7.2%)	1115 (27.2%)	< 0.001	< 0.001
Glycopeptides	7144 (11.6%)	4280 (7.9%)	2864 (39.4%)	< 0.001	3526 (15.0%)	2182 (11.2%)	1344 (32.8%)	< 0.001	< 0.001
Penicillins	3389 (5.5%)	3082 (5.7%)	307 (4.2%)	< 0.001	211 (0.9%)	150 (0.8%)	61 (1.5%)	< 0.001	< 0.001
Quinolones	3926 (6.4%)	1773 (3.3%)	2153 (29.6%)	< 0.001	4369 (18.5%)	2845 (14.6%)	1524 (37.2%)	< 0.001	< 0.001

Characteristics are summarized as the median [IQR], or n (%).

*ICU* intensive care unit, *APACHE* Assessment and Chronic Health Evaluation, *SAPS* Simplified Acute Physiology Score, *MPM* Mortality Probability Model, *SOFA* Sequential Oran Failure Assessment.

*P value for difference between cases with and without in-hospital mortality.

^†^*P* value for difference between hospital S and hospital G.

There was a significant difference in the composition of patients admitted to the ICUs of the two cohorts. Hospital G had a higher rate of admission due to surgical factors than hospital S (51.8% vs. 35.8%, *P* < 0.001). The rate of cardiac arrest within 24 h of ICU admission was significantly higher in hospital G than in hospital S (5.2% vs. 1.3%; *P* < 0.001). In both hospitals, positive bacterial culture results were associated with a higher mortality rate than culture-negative or nonmicrobiological test prescriptions, but the bacterial culture positive rates in each cohort showed a statistically significant difference (3.4% in hospital S vs. 11.3% in hospital G; *P* < 0.001).

The proportions of departments at the time of ICU admission in each cohort also differed significantly (Table S1; *P* < 0.001). In hospital S, Departments of Cardiology, Neurology, and Cardiac Surgery had the highest proportions of inpatients (53.06%, 18.58%, and 17.38%, respectively), while in hospital G, the values for the Departments of Cardiology, Neurosurgery, and Thoracic Surgery were 25.33%, 20.5%, and 9.3%, respectively.

Table 2 shows that the conventional scoring systems had various predictive powers for each hospital and different study periods. For each study period, the highest AUROC values were 0.757 (95% CI, 0.744–0.769; SAPS II in 2005–2010), 0.777 (95% CI, 0.767–0.788; SAPS III in 2011–2015), and 0.808 (95% CI, 0.798–0.817; APACHE II in 2016–2020) in hospital S and 0.805 (95% CI, 0.790–0.819; APACHE III in 2005–2010), 0.789 (95% CI, 0.776–0.802; APACHE III and 95% CI, 0.773–0.799; SAPS III in 2011–2015), 0.831 (95% CI, 0.820–0.842; SAPS III in 2016–2020) in hospital G. During the entire study period, SAPS III showed the highest AUROC value (0.773 [95% CI, 0.766–0.779]) in hospital S, followed by SAPS II (0.766 [95% CI, 0.759–0.772]) and APACHE III (0.755 [95% CI, 0.747–0.762]). However, during certain periods, such as 2016–2020, APACHE II had the highest AUROC. Similarly, in hospital G, the best predictive model differed by study period. This performance was calculated by applying an optimal cutoff value (the point with the highest Youden’s index^25^) with the highest AUROC, and we found that these cutoff values increased over time for most models.

**Table 2:** Performance metrics for the conventional scoring systems by hospital and study period.

	Hospital S in 2005–2010				Hospital S in 2011–2015				Hospital S in 2016–2020				Hospital S in Total period			
	AUROC (95% CI)	Cutoff	Accuracy	F1 score	AUROC (95% CI)	Cutoff	Accuracy	F1 score	AUROC (95% CI)	Cutoff	Accuracy	F1 score	AUROC (95% CI)	Cutoff	Accuracy	F1 score
APACHE II	0.691 (0.678–0.704)	15	0.686	0.318	0.721 (0.709–0.732)	19	0.794	0.376	0.808 (0.798–0.817)	17	0.754	0.379	0.738 (0.731–0.745)	17	0.747	0.356
APACHE III	0.720 (0.706–0.735)	64	0.793	0.405	0.747 (0.735–0.759)	70	0.821	0.441	0.798 (0.787–0.809)	68	0.813	0.429	0.755 (0.747–0.762)	69	0.826	0.436
SAPS II	0.757 (0.744–0.769)	38	0.776	0.412	0.770 (0.759–0.781)	44	0.799	0.427	0.793 (0.782–0.804)	47	0.831	0.441	0.766 (0.759–0.772)	45	0.822	0.430
SAPS III	0.756 (0.743–0.768)	53	0.730	0.388	0.777 (0.767–0.788)	56	0.793	0.423	0.781 (0.771–0.791)	56	0.770	0.378	0.773 (0.766–0.779)	56	0.793	0.401
MPM0 II	0.574 (0.560–0.588)	1	0.719	0.290	0.547 (0.534–0.560	1	0.768	0.291	0.540 (0.526–0.553)	3	0.791	0.267	0.477 (0.469–0.485)	3	0.766	0.222
MPM0 III	0.612 (0.598–0.626)	1	0.772	0.317	0.563 (0.550–0.577)	1	0.795	0.297	0.504 (0.490–0.518)	4	0.862	0.226	0.553 (0.545–0.561)	1	0.787	0.285
SOFA	0.647 (0.634–0.660)	4	0.600	0.296	0.693 (0.682–0.705)	5	0.633	0.312	0.784 (0.774–0.793)	6	0.681	0.343	0.706 (0.699–0.712)	5	0.625	0.307
Quick SOFA	0.526 (0.513–0.539)	0	0.754	0.216	0.513 (0.501–0.526)	3	0.825	0.164	0.555 (0.542–0.567)	3	0.770	0.229	0.512 (0.505–0.520)	3	0.816	0.173
Pitt bacteremia score	0.537 (0.526–0.549)	2	0.627	0.238	0.585 (0.573–0.596)	2	0.613	0.260	0.607 (0.595–0.619)	8	0.866	0.270	0.576 (0.569–0.583)	2	0.603	0.240

	Hospital G in 2005–2010				Hospital G in 2011–2015				Hospital G in 2016–2020				Hospital G in Total period			
	AUROC (95% CI)	Cutoff	Accuracy	F1 score	AUROC (95% CI)	Cutoff	Accuracy	F1 score	AUROC (95% CI)	Cutoff	Accuracy	F1 score	AUROC (95% CI)	Cutoff	Accuracy	F1 score
APACHE II	0.792 (0.777–0.806)	17	0.684	0.436	0.784 (0.771–0.796)	19	0.696	0.473	0.804 (0.793–0.816)	21	0.762	0.476	0.792 (0.785–0.800)	19	0.711	0.456
APACHE III	0.805 (0.790–0.819)	64	0.696	0.456	0.789 (0.776–0.802)	69	0.720	0.487	0.814 (0.803–0.826)	69	0.745	0.488	0.803 (0.795–0.810)	69	0.740	0.484
SAPS II	0.781 (0.766–0.797)	40	0.751	0.465	0.784 (0.771–0.797)	42	0.696	0.482	0.828 (0.817–0.839)	47	0.743	0.501	0.792 (0.784–0.799)	44	0.733	0.480
SAPS III	0.786 (0.771–0.802)	55	0.739	0.472	0.789 (0.773–0.799)	56	0.718	0.493	0.831 (0.820–0.842)	60	0.756	0.508	0.798 (0.791–0.806)	56	0.716	0.481
MPM0 II	0.621 (0.602–0.640)	2	0.571	0.322	0.595 (0.578–0.611)	3	0.783	0.325	0.611 (0.595–0.627)	3	0.800	0.320	0.608 (0.598–0.617)	3	0.797	0.318
MPM0 III	0.677 (0.658–0.696)	3	0.750	0.393	0.654 (0.638–0.671)	3	0.744	0.411	0.679 (0.664–0.695)	3	0.768	0.409	0.670 (0.660–0.679)	3	0.755	0.405
SOFA	0.760 (0.745–0.775)	7	0.672	0.418	0.772 (0.759–0.784)	8	0.656	0.448	0.783 (0.772–0.795)	8	0.639	0.422	0.771 (0.764–0.779)	8	0.671	0.432
Quick SOFA	0.641 (0.626–0.656)	2	0.577	0.354	0.618 (0.604–0.632)	2	0.434	0.355	0.677 (0.665–0.690)	3	0.654	0.386	0.634 (0.626–0.642)	2	0.433	0.342
Pitt bacteremia score	0.736 (0.720–0.752)	3	0.611	0.404	0.733 (0.720–0.746)	4	0.637	0.433	0.720 (0.707–0.733)	4	0.572	0.385	0.727 (0.719–0.736)	4	0.638	0.410

*AUROC* area under the receiver operating characteristic curve, *APACHE* Assessment and Chronic Health Evaluation, *SAPS* Simplified Acute Physiology Score. *MPM* Mortality Probability Model, *SOFA* Sequential Oran Failure Assessment.

### ML model

**Figure 2:**
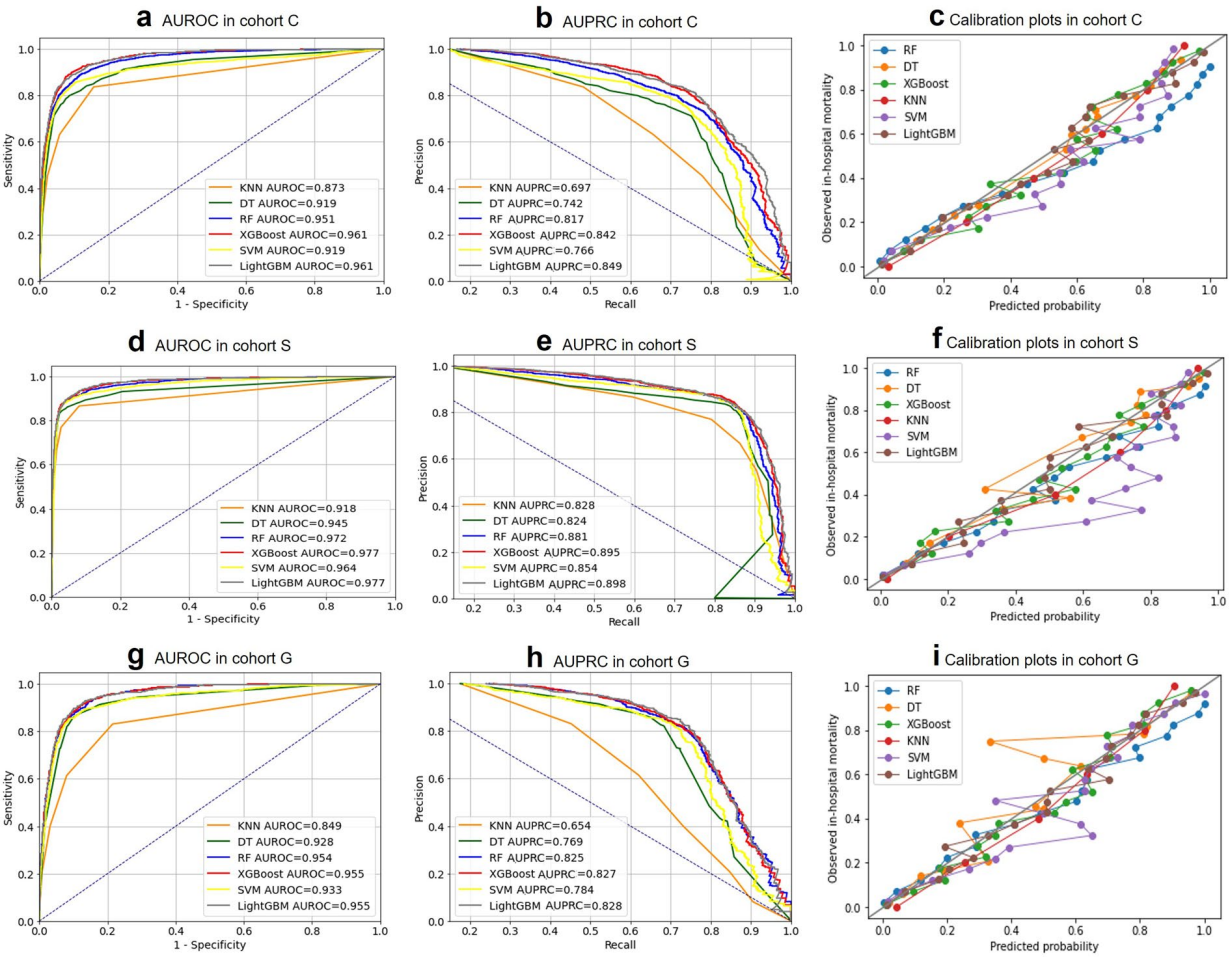
Comparison of machine learning-based in-hospital mortality prediction models. *AUROC* area under the receiver operating characteristic curve, *AUPRC* area under the precision-recall curve, *KNN* K-nearest neighbor, *DT* decision tree, *RF* random forest, *XGBoost* eXtreme gradient boosting, *LightGBM* light gradient boosting, SVM support vector machine. The calibration plots show the agreement of between predicted probability and observed in-hospital mortality. The black line at 45 degrees indicates perfect calibration where the predicted and observed probabilities are equal.

Table 3 summarizes the performance of ML algorithms for each dataset. For cohort C to equally reflect the features of the two hospitals, the cases from the hospital S dataset were randomly selected and combined with the same size as the cases from the hospital G dataset. For all variables, there were no significant differences between the randomly selected dataset and the full data of hospital S (Table S2). For cohort S and cohort G, ML was performed using the datasets of hospital S and hospital G, respectively. For cohort C, the XGBoost and LightGBM classifiers showed the highest AUROC value of 0.961 (95% CI, 0.957–0.965 for both), and the LightGBM classifier had the highest F1 score of 0.765. The classifiers that performed best with cohort S and cohort G were different. For cohort S, the XGBoost classifier achieved the highest values in both the F1 score (0.840) and AUROC (0.977; 95% CI, 0.973–0.980), and for cohort G, the LightGBM classifier showed both the highest F1 score (0.762) and the highest AUROC (0.955; 95% CI, 0.950–0.961). All ML algorithms (except for KNN with cohort C and cohort G) showed outstanding discriminant performance with an AUROC of 0.9 or higher^26^ and had superior performance to the conventional scoring systems in terms of the AUROC, accuracy, and F1 score. Figure 2 present the AUROCs, AUPRCs, and calibration plots of cohort C (Fig. 2a–c), cohort S (Fig. 2d–f), and cohort G (Fig. 2g–i) for in-hospital mortality. In all three models, the LightGBM classifier achieved the best AUPRC.

**Table 3:** Performance metrics for the machine learning algorithms with the test set for each cohort.

Cohort C	AUROC (95% CI)	Accuracy	F1 score
К-nearest neighbor (KNN)	0.873 (0.864–0.882)	0.899	0.573
Decision tree (DT)	0.919 (0.911–0.926)	0.922	0.731
Random forest (RF)	0.951 (0.846–0.956)	0.923	0.731
eXtreme gradient boosting (XGBoost)	0.961 (0.957–0.965)	0.932	0.758
Light gradient boosting (LightGBM)	0.961 (0.957–0.965)	0.933	0.765
Support vector machine (SVM)	0.919 (0.911–0.928)	0.921	0.690
Artificial neural network (ANN)	0.950 (0.941–0.959)	0.931	0.751

Cohort S	AUROC (95% CI)	Accuracy	F1 score
К-nearest neighbor (KNN)	0.918 (0.910–0.925)	0.947	0.752
Decision tree (DT)	0.945 (0.938–0.952)	0.960	0.830
Random forest (RF)	0.972 (0.969–0.976)	0.962	0.830
eXtreme gradient boosting (XGBoost)	0.977 (0.973–0.980)	0.963	0.840
Light gradient boosting (LightGBM)	0.977 (0.974–0.981)	0.962	0.839
Support vector machine (SVM)	0.964 (0.959–0.969)	0.957	0.807
Artificial neural network (ANN)	0.970 (0.962–0.978)	0.961	0.832

Cohort G	AUROC (95% CI)	Accuracy	F1 score
К-nearest neighbor (KNN)	0.849 (0.836–0.862)	0.869	0.518
Decision tree (DT)	0.928 (0.919–0.936)	0.903	0.725
Random forest (RF)	0.954 (0.949–0.959)	0.914	0.725
eXtreme gradient boosting (XGBoost)	0.955 (0.950–0.960)	0.916	0.750
Light gradient boosting (LightGBM)	0.955 (0.950–0.961)	0.918	0.762
Support vector machine (SVM)	0.933 (0.925–0.941)	0.913	0.749
Artificial neural network (ANN)	0.951 (0.939–0.963)	0.911	0.729

*AUROC* area under the receiver operating characteristic curve, *CI* Confidence interval.

The results of external validation are described in Table S3. We validated the ML model obtained from cohort S with the dataset of cohort G and vice versa. The AUROCs of the models deteriorated to 0.654 (for the XGBoost classifier; 95% CI, 0.648–0.662) and 0.586 (for the LightGBM classifier; 95% CI, 0.581–0.591).

### Critical variables with feature importance plots and SHAP values for predicting in-hospital mortality

**Figure 3:**
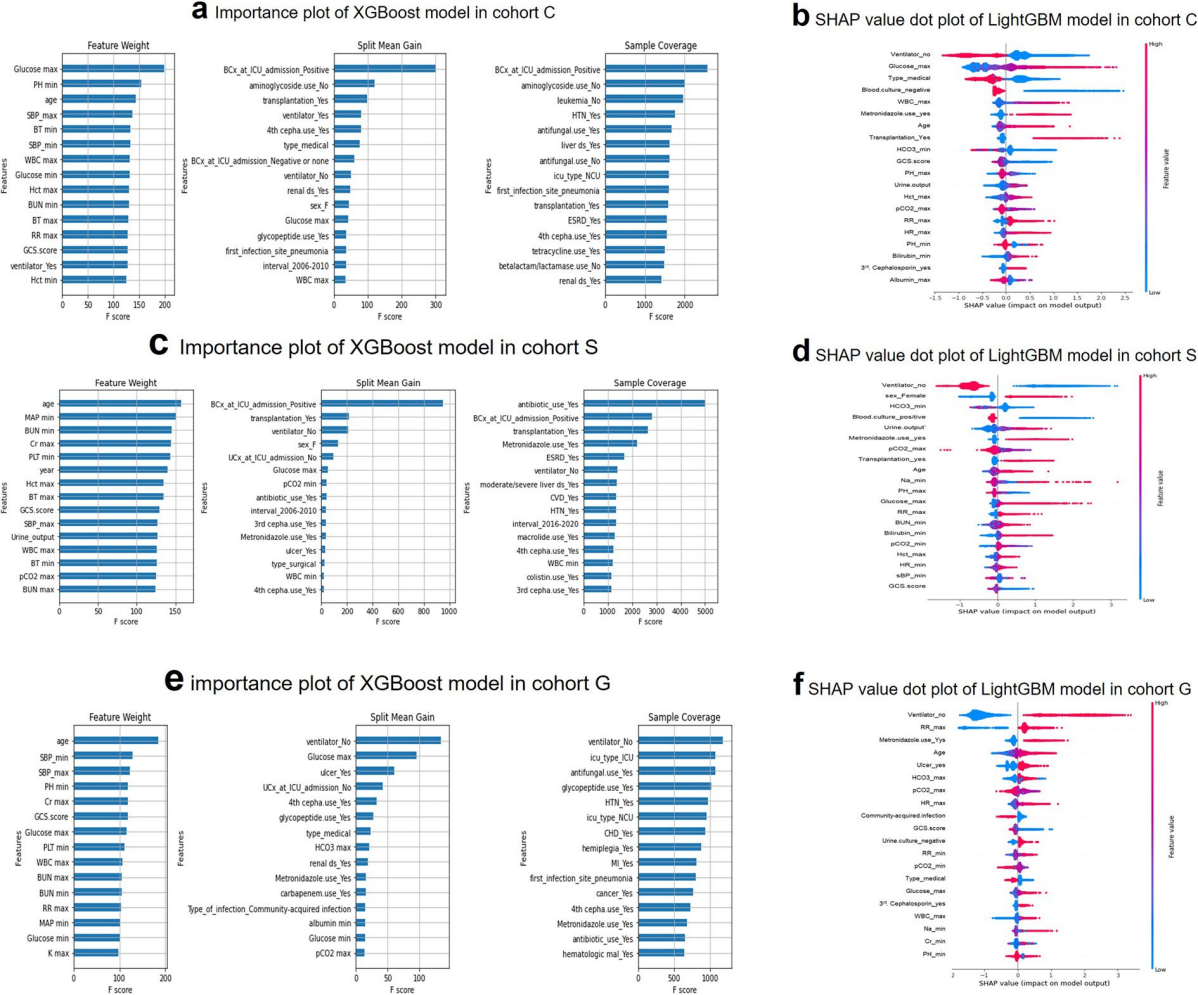
Critical variables with feature importance plots and SHAP values for predicting in-hospital mortality. Critical variables with feature importance plots and SHAP values for predicting in-hospital mortality. *XGBoost* extreme gradient boost, *LightGBM* light gradient boosting machine, *SHAP* Shapley additive explanation. (**a**) Feature importance including feature weight, mean gain and coverage of XGBoost and (**b**) SHAP value summary dot plot of the LightGBM-based prediction model in cohort C. (**c**) Feature importance including feature weight, mean gain and coverage of XGBoost and (**d**) SHAP value summary dot plot of the LightGBM-based prediction model in cohort S. (**e**) Feature importance including feature weight, mean gain and coverage of XGBoost and (**f**) SHAP value summary dot plot of the LightGBM-based prediction model in cohort G. The color of the SHAP dot represents the value of the feature, and the location of the dot on the X axis represents its SHAP value. Red dots indicate higher values or affirmative responses (for binary features), and blue dots indicate the opposite. A positive SHAP value indicates that the variables increase the likelihood of in-hospital mortality.

The top predictors for in-hospital mortality obtained in each cohort are described in Fig. 3 (a–d, and d-f represent cohort C, cohort S, and cohort G, respectively). Figure 3a,c,e show the most important features calculated using the XGBoost models for in-hospital mortality. In cohort C, maximum serum glucose and positive blood culture results; in cohort S, patient age, positive blood culture results, and history of antibiotic use; and in cohort G, patient age and ventilator application were the top predictors for in-hospital mortality. SHAP analysis of the LightGBM models was also performed to investigate which individual variables impact in-hospital mortality prediction. For cohort C (Fig. 3b), nonventilator use, maximum serum glucose, and admission for medical reasons were the top indicators associated with in-hospital mortality. For cohort S and cohort G, there was a difference in the top variables between the models trained on datasets from different hospitals. Female sex and positive blood culture results were more important in cohort C (Fig. 3d), while a history of ulcers and community-acquired infection were more important in cohort G (Fig. 3f).

## Discussion

Many hospitals and clinics already use EHR systems, and some of them have data extraction programs for analyzing big data that are accumulated in real time. ML analysis with these tools is much more convenient to use than traditional manual scoring systems, leverages the large amount of data already collected from EHRs to provide better predictive performance, and can even be automated to support clinical decision-making^15,27,28^. In this study, we demonstrated the ability of various ML algorithms to predict in-hospital mortality of ICU inpatients using EHR data and achieved improved performance compared with conventional predictive models.

Several previous studies had compared the predictive performance in prognosis of ICU patients, and various machine learning algorithms, including ANN, RF, and XGBoost were showed superior performance than conventional models^15,16,18,29,30^. In our study, all of the conventional models showed relatively low F1 scores (below 0.51), which is an informative index that evaluates binary classifiers on imbalanced datasets^22,23^. This might be because the conventional scoring systems failed to overcome the fundamental imbalance in clinical outcomes, where the in-hospital mortality rates of ICU inpatients included in cohort S and cohort G were 11.8% and 17.4%, respectively. In contrast, the F1 scores were largely improved in the ML models. In particular, the models trained with cohort S (the highest F1 score was 0.840), which consisted only of the data from hospital S, had a better fit than with cohort C (the highest F1 score was 0.765), which was constructed by combining data from the two hospitals. The conventional scoring system was modeled as a logistic regression method that could be affected by multicollinearity between variables, and the power of the model is expected to be reduced because it assumes that the relationship between all independent variables is linear^31^. ML-based models have inherent advantages for correcting nonlinear relationships and the multicollinearity of independent variables, and flexible updates are possible even when new clinical data are added. Thus, it has shown better performance in previous reports than models based on the conventional logistic regression method^15,16,32^.

Conventional models had moderate accuracy in predicting in-hospital mortality with our data, which is consistent with prior reports^1,3–7^. In particular, SAPS III and APACHE III had the highest AUROC among these models during the entire study period. Interestingly, the Pitt bacteremia score showed relatively higher performance with cohort G than with cohort S, which might be because the rate of cardiac arrest within 24 h of ICU admission, a variable included in the Pitt bacteremia score, was higher in hospital G than in hospital S. In our data, the optimal cutoff values with the highest AUROC increased over time in most models, possibly due to improved patient prognosis with the advances in health care systems and the extension of life expectancy. We applied updated optimal cutoffs for each study period to avoid the performance deterioration of conventional models seen in previous studies^10,12,33–36^.

We also demonstrated the top variables that had the largest impact on the predictive performance of the ML models in two university hospitals with differences in patient demographics, in-hospital mortality rate, and organizational practices. To predict in-hospital mortality in cohort C, ventilator application, glucose, type of admission, and blood culture results were selected as top predictors. However, the algorithm selected different features from the local data for cohort S and cohort G as the most predictive variables. ML models reflect more of the characteristics of each cohort and have a nontransferable and specific natures, as shown in the deteriorated external validation results of this study. Because each center has fundamentally different patient composition and characteristics, predictive models can be made more robust using ML approach trained on data from individual hospitals, but this inevitably leads to degrade performance on external data sets. Also in our study, algorithms such as LightGBM and XGBoost that performed best in the test set showed relatively more performance degradation in the external validation set. ML approach may have limitations in standardizing clinical predictive indicators or comparing the quality of care between centers^18^. In contrast, conventional scoring systems have relatively inferior predictive performance, but have strengths in standardization and center-to-center comparisons. Therefore, the use of ML models in conjunction with conventional scoring systems can provide more useful information for predicting the prognosis of critically ill patients or for comparing and evaluating ICUs.

Our study has several limitations. First, the models may not be generalized to other hospitals due to the single-country design of the study. In addition, residual confounding such as patient’s racial and socioeconomic status could act as algorithm bias. However, considering that the performance of the predictive model deteriorates over time, we attempted to demonstrate the importance of a system that can be updated continuously and can reflect the features of individual centers. Second, patients without demographic data or with more than 20% missing data were excluded from this study. Missing or inaccurate data may have affected the predictive power of the models. However, we attempted to minimize the impact of missing by using large data. It is expected that the improvement of EHR and data extraction tools can solve this problem in the near future.

## Conclusion

We demonstrated the importance of an updatable predictive system that can reflect the characteristics of individual ICUs. Improvements in information technology and ML have opened new opportunities for analyzing clinical information. These techniques can be utilized to automatically and accurately collect patient data to support clinical decision-making using complex algorithms. Our study, employing models trained and validated on real-world data with two different cohorts, showed that ML-based models have accurate predictive power in the ICUs. In addition, conventional models have strengths in standardization and center-to-center comparisons. Using ML models in conjunction with conventional scoring systems can provide more useful information for predicting the prognosis of critically ill patients.

## Supplementary Information

Supplementary Tables.

## Data Availability

The datasets generated and analysed during the current study are not publicly available but are available from the corresponding author on reasonable request.
