# Supplementary material for: Mortality prediction of patients in intensive care units using machine learning algorithms based on electronic health records
Source: Sci Rep. 2022 May 3;12:7180. doi: 10.1038/s41598-022-11226-4 (PMC9065110; doi:10.1038/s41598-022-11226-4)
Supplement: Supplementary file 1 — Supplementary Tables. [file 41598_2022_11226_MOESM1_ESM.docx]

Table S1. Comparison of inpatient departments at the time of admission to the ICU by cohort

|  | Hospital S | Hospital G |
| --- | --- | --- |
| Admitted department | (N=61589) | (N=23557) |
| Cardiology | 32681 (53.06%) | 5967 (25.33%) |
| Cardiovascular Surgery | 10705 (17.38%) | 1936 (8.22%) |
| Colorectal and Anal Surgery | 51 (0.08%) | 369 (1.57%) |
| Endocrinology | 6 (0.01%) | 103 (0.44%) |
| Gastroenterology | 686 (1.11%) | 1212 (5.14%) |
| Gastrointestinal surgery | 29 (0.05%) | 57 (0.24%) |
| General surgery | 294 (0.48%) | 1071 (4.55%) |
| Hematology | 797 (1.29%) | 145 (0.62%) |
| Hepatobiliary and Pancreatic Surgery | 22 (0.04%) | 309 (1.31%) |
| Infectious Disease | 434 (0.7%) | 598 (2.54%) |
| Nephrology | 211 (0.34%) | 558 (2.37%) |
| Neurology | 11443 (18.58%) | 666 (2.83%) |
| Neurosurgery | 1192 (1.94%) | 4829 (20.5%) |
| Oncology | 544 (0.88%) | 304 (1.29%) |
| Orthopedics | 39 (0.06%) | 583 (2.47%) |
| Otorhinolaryngology | 29 (0.05%) | 156 (0.66%) |
| Rehabilitation | 11 (0.02%) | 291 (1.24%) |
| Respiratory | 1277 (2.07%) | 1429 (6.07%) |
| Rheumatology | 41 (0.07%) | 23 (0.1%) |
| Thoracic Surgery | 223 (0.36%) | 2190 (9.3%) |
| Thyroid Endocrine Surgery | 2 (0%) | 97 (0.41%) |
| Urosurgery | 32 (0.05%) | 98 (0.42%) |
| Other | 840 (1.36%) | 566 (2.4%) |

Table S2. Comparison of the overall data for hospital S with the randomly selected data for cohort C

|  | Hospital S | Randomly selective for Cohort C | *p* |
| --- | --- | --- | --- |
| Admission variables (obtained within 24 hours of ICU admission) | (N=61589) | (N=23557) |  |
| Age, years | 67.0 [57.0-74.0] | 66.0 [57.0-74.0] | 0.491 |
| Sex |  |  | 0.792 |
| Female | 22744 (36.9%) | 8723 (37.0%) |  |
| Male | 38845 (63.1%) | 14834 (63.0%) |  |
| Type of admission |  |  | 0.962 |
| Medical | 39560 (64.2%) | 15136 (64.3%) |  |
| Surgical | 22029 (35.8%) | 8421 (35.7%) |  |
| Year of admission |  |  | 0.926 |
| 2006-2010 | 16531 (26.8%) | 6293 (26.7%) |  |
| 2011-2015 | 20945 (34.0%) | 8016 (34.0%) |  |
| 2016-2020 | 24113 (39.2%) | 9248 (39.3%) |  |
| Underlying comorbidities |  |  |  |
| Cancer | 5137 (8.3%) | 2016 (8.6%) | 0.313 |
| Cerebrovascular diseases | 14373 (23.3%) | 5394 (22.9%) | 0.177 |
| Diabetes mellitus | 16696 (27.1%) | 6414 (27.2%) | 0.734 |
| Hypertension | 28407 (46.1%) | 10905 (46.3%) | 0.665 |
| Chronic pulmonary diseases | 1832 (3.0%) | 702 (3.0%) | 0.985 |
| Hemiplegia | 2041 (3.3%) | 802 (3.4%) | 0.524 |
| Liver diseases | 2080 (3.4%) | 782 (3.3%) | 0.692 |
| Myocardial infarction | 11682 (19.0%) | 4470 (19.0%) | 0.988 |
| Renal diseases | 3462 (5.6%) | 1350 (5.7%) | 0.546 |
| Ulcer | 1168 (1.9%) | 458 (1.9%) | 0.669 |
| Transplantation | 766 (1.2%) | 312 (1.3%) | 0.364 |
| Ventilator use | 10537 (17.1%) | 4066 (17.3%) | 0.606 |
| Vasopressor use | 21448 (34.8%) | 8252 (35.0%) | 0.579 |
| Cardiac arrest | 809 (1.3%) | 316 (1.3%) | 0.775 |
| Infection on ICU admission |  |  |  |
| Site of infection |  |  | 0.980 |
| Multiple sites | 843 (1.4%) | 330 (1.4%) |  |
| Pneumonia | 428 (0.7%) | 157 (0.7%) |  |
| Bloodstream | 251 (0.4%) | 95 (0.4%) |  |
| Urinary tract | 503 (0.8%) | 204 (0.9%) |  |
| CNS | 3 (0.0%) | 2 (0.0%) |  |
| Abdomen | 27 (0.0%) | 12 (0.1%) |  |
| None | 59521 (96.6%) | 22751 (96.6%) |  |
| Infection type |  |  | 0.775 |
| Community-acquired infection | 1960 (3.2%) | 752 (3.2%) |  |
| Nosocomial infection | 3916 (6.4%) | 1529 (6.5%) |  |
| Antibiotic use at ICU admission (may be multiple) | 28255 (45.9%) | 10788 (45.8%) | 0.837 |
| 3rd-generation cephalosporins | 6788 (11.0%) | 2534 (10.8%) | 0.274 |
| 4th-generation cephalosporins | 450 (0.7%) | 178 (0.8%) | 0.737 |
| Beta lactam/beta lactamase inhibitors | 8744 (14.2%) | 3363 (14.3%) | 0.777 |
| Carbapenems | 2300 (3.7%) | 873 (3.7%) | 0.860 |
| Glycopeptides | 7144 (11.6%) | 2700 (11.5%) | 0.582 |
| Penicillins | 3389 (5.5%) | 1314 (5.6%) | 0.679 |
| Quinolones | 3926 (6.4%) | 1488 (6.3%) | 0.769 |

Table S3. Performance metrics for the machine learning algorithms with external validation

| Validated with hospital G with models trained with cohort S | AUROC (95% CI) | Accuracy | F1 score |
| --- | --- | --- | --- |
| Decision tree (DT) | 0.672 (0.665-0.679) | 0.624 | 0.292 |
| Random forest (RF) | 0.566 (0.559-0.573) | 0.615 | 0.220 |
| eXtreme gradient boosting (XGBoost) | 0.654 (0.648-0.662) | 0.649 | 0.330 |
| Light gradient boosting (LightGBM) | 0.663 (0.656-0.670) | 0.638 | 0.347 |
| Support vector machine (SVM) | 0.602 (0.595-0.610) | 0.625 | 0.275 |
| Artificial neural network (ANN) | 0.649 (0.642-0.655) | 0.663 | 0.323 |
|  |  |  |  |
| Validated with hospital S with models trained with cohort G | **AUROC (95% CI)** | **Accuracy** | **F1 score** |
| Decision tree (DT) | 0.559 (0.555-0.563) | 0.632 | 0.049 |
| Random forest (RF) | 0.660 (0.656-0.664) | 0.790 | 0.124 |
| eXtreme gradient boosting (XGBoost) | 0.619 (0.615-0.624) | 0.831 | 0.122 |
| Light gradient boosting (LightGBM) | 0.586 (0.581-0.591) | 0.814 | 0.118 |
| Support vector machine (SVM) | 0.345 (0.340-0.350) | 0.754 | 0.055 |
| Artificial neural network (ANN) | 0.506 (0.503-0.509) | 0.831 | 0.102 |

*AUROC* area under the receiver operating characteristic curve, *CI* confidence interval

Table S4. Feature importance scores in Fig. 3.

| Feature importance score in cohort C | | |  | Feature importance score in cohort S | | |  | Feature importance score in cohort G | | |
| --- | --- | --- | --- | --- | --- | --- | --- | --- | --- | --- |
| Feature Weight | Split Mean Gain | Sample Coverage |  | Feature Weight | Split Mean Gain | Sample Coverage |  | Feature Weight | Split Mean Gain | Sample Coverage |
| 198 | 300 | 2576 |  | 156 | 953 | 5018 |  | 183 | 134 | 1152 |
| 154 | 117 | 1998 |  | 149 | 220 | 2807 |  | 127 | 95 | 1056 |
| 142 | 96 | 1957 |  | 144 | 207 | 2649 |  | 122 | 62 | 1051 |
| 136 | 78 | 1739 |  | 142 | 133 | 2211 |  | 118 | 42 | 1000 |
| 132 | 78 | 1663 |  | 141 | 93 | 1667 |  | 116 | 32 | 944 |
| 132 | 75 | 1620 |  | 138 | 50 | 1404 |  | 116 | 27 | 933 |
| 131 | 57 | 1609 |  | 133 | 40 | 1316 |  | 114 | 22 | 904 |
| 130 | 51 | 1587 |  | 133 | 33 | 1316 |  | 109 | 21 | 854 |
| 130 | 48 | 1577 |  | 128 | 33 | 1316 |  | 105 | 19 | 798 |
| 129 | 42 | 1576 |  | 127 | 33 | 1316 |  | 104 | 17 | 787 |
| 128 | 39 | 1533 |  | 126 | 33 | 1298 |  | 104 | 17 | 747 |
| 126 | 36 | 1532 |  | 124 | 30 | 1211 |  | 103 | 15 | 708 |
| 126 | 36 | 1489 |  | 124 | 27 | 1193 |  | 100 | 14 | 674 |
| 126 | 36 | 1467 |  | 124 | 23 | 1123 |  | 100 | 14 | 640 |
| 124 | 33 | 1402 |  | 123 | 20 | 1105 |  | 96 | 14 | 629 |
